# Laparoscopic Versus Robotic Ventral Hernia Repair With Intraperitoneal Mesh: A Systematic Review and Meta-Analysis Comparing the Perioperative Outcomes Randomised Controlled Trials

**DOI:** 10.3389/jaws.2024.13809

**Published:** 2025-01-22

**Authors:** Anurag Singh, Wei H. Toh, Nada Elzahed, Goldie Khera, Mirza K. Baig, Andrei Mihailescu, Muhammad S. Sajid

**Affiliations:** ^1^ Department of General Surgery, Tameside General Hospital, Ashton-Under-Lyne, United Kingdom; ^2^ Department of Gastrointestinal Surgery, Royal Sussex County Hospital, Brighton, United Kingdom; ^3^ Department of Colo-Rectal Surgery, Worthing Hospital, Worthing, United Kingdom

**Keywords:** laparoscopic ventral hernia repair, robotic ventral hernia repair, perioperative outcomes, cost comparison, IPOM

## Abstract

**Objective:**

The objective of this meta-analysis is to compare the perioperative surgical outcomes and cost-effectiveness of robotic ventral hernia repair (RVHR) versus laparoscopic ventral hernia repair (LVHR) with intraperitoneal mesh.

**Methods:**

Randomised control trials (RCTs) reporting perioperative outcomes and costs in patients undergoing RVHR versus LVHR were selected from medical electronic databases and meta-analysis was conducted in accordance with the guidelines of the Cochrane Collaboration using statistical software RevMan version 5.

**Results:**

Four RCTs on 337 patients reporting perioperative outcomes and cost comparison were included. In the random effect model analysis, the duration of operation was shorter, and cost was lower in the LVHR group but with significant statistical heterogeneity [standardized mean difference (SMD) −48.07, 95%, CI (−78.06, −18.07), Z = 3.14, P = 0.002], [SMD 0.82, 95%, CI (−1.48, −0.16), Z = 2.45, P = 0.01]. However, the variables of hernia recurrence and surgical site complications were statistically similar in both groups without any statistical heterogeneity among the included studies [Risk Ratio (RR) 1.05, 95%, CI (0.22, 4.99), Z = 0.06, P = 0.95], [RR 0.85, 95%, CI (0.48, 1.50), Z = 0.55, P = 0.58].

**Conclusion:**

This systematic review demonstrates that RVHR does not offer any superiority among the compared perioperative variables (Duration of operation, hernia recurrence and surgical site complications) and it is not cost-effective when compared to LVHR. Due to the paucity of the RCTs and significant heterogeneity among the compared variables, a major multi-centre RCT is needed to validate these findings.

## Introduction

A ventral hernia is one of the most common presentations among the patients encountered by the surgeons and the general practitioner [1]. In the UK alone around 100,000 abdominal wall hernia surgeries are repaired annually [2] which includes a significant proportion of ventral hernias (VH). The operative management of VH can be challenging due to the diversity of surgical approaches (open repair, laparoscopic repair, robotic repair), techniques of mesh fixation, size of the defect and implantation of a wide variety of biological or synthetic meshes necessary to achieve desired outcomes of minimum hernia recurrence risk and other post-operative complications such as surgical site infection, haemorrhage and enterocutaneous fistula [3]. Prior to the 1990s, VH repairs were primarily done through an open approach [4]. With the advent of the laparoscopic approach, surgeons utilised this approach to reduce the risk of hernia recurrence and postoperative surgical site infections with better health-related quality of life. Nonetheless, the laparoscopic approach is associated with a few drawbacks, primarily poor views due to intra-abdominal adhesions and reduced manoeuvrability of laparoscopic instruments [5].

The first documented use of the robotic approach for surgical procedures was reported in the year 2000 [6]. The use of a robotic approach for the surgical resections of urological malignancies and gynaecological malignancies has been a common practice in the last two decades with variable and diverse outcomes [7, 8]. Published studies have reported favourable outcomes for robotic ventral hernia repair (RVHR) compared to the relatively conventional approach of laparoscopic ventral hernia repair (LVHR) There are several studies reported in the literature demonstrating the superiority of the robotic approach for ventral hernia repair over the laparoscopic approach [7–9]. Due to the extra cost involved in performing the robotic procedures, there has always existed a debate among hernia surgeons about the effectiveness of the robotic approach. Primary variables such as duration of operation, blood loss, bowel injury, length of hospital stay, hernia recurrence, post-operative wound-related and systemic complications may well be similar in RVHR versus LVHR but secondary or tertiary variables like extra cost of the procedure and similar postoperative health-related quality of life seem to be a limiting factor in the use of robotic approach in the management of VH. The objective of this meta-analysis is to compare the perioperative surgical outcomes and cost-effectiveness of RVHR versus LVHR and report.

## Methods

### Data Sources and Literature Search Technique

This meta-analysis has been registered with the research registry reviewregistry1726. Electronic databases PubMed, EMBASE, MEDLINE and the Cochrane Library were reviewed and carefully searched. Relevant articles were identified with the use of MeSH terms (Robotic hernia repair, Laparoscopic hernia repair, Ventral hernia repair) and Boolean operators (AND, OR) and PICO approach was used to systematically refine and narrow down the search results. The references were further searched to identify the relevant articles for a detailed analysis.

### Trial Selection

The inclusion criteria for the systematic review was the randomised control trials (RCTs) comparing RVHR against LVHR, reporting perioperative outcomes and cost analysis. All trials regardless of their language of publication and number of recruited patients were deemed suitable for inclusion.

### Data Collection and Management

The published data was searched and collected by authors independently on a pre-planned standard data extraction sheet. The collected data was scrutinized involving all authors to detect any discrepancy and a mutual agreement was reached about accuracy. The main variables for data collection included were the list of published authors, the country where the RCT was conducted, year of publication, demographic details of the study population, hernia recurrence numbers, duration of operation, surgical site infections, wound seroma, wound break down, delayed wound healing, cost of the laparoscopic procedure and the cost of the robotic procedure.

### Evidence Synthesis Using RevMan Statistical Software

RevMan version 5.4 (Review Manager 5.4, The Nordic Cochrane Centre, Copenhagen, Denmark) was used for the statistical analysis [10] of the data. In order to present the summated outcome of continuous variables such as cost of the procedure and duration of operation; the standardised mean difference (SMD) was used, and the risk ratio was used to present the summated outcomes of dichotomous data (Wound complication and hernia recurrence). The SMD and RR were calculated and presented with a 95% confidence interval (CI) under the random-effects model analysis [11, 12]. A forest plot was used for the graphical presentation of the results. The statistical heterogeneity was calculated by computing the chi^2^ test, with significance set at P < 0.05 whereas the quantification of the heterogeneity was tested using the I^2^ test with a maximum value of 30 per cent identifying low heterogeneity [13]. For the calculation of the SMD, the inverse-variance method was used and for the calculation of the risk ratio, the Mantel- Haenszel method was used under the random effect model analysis [14, 15]. If standard deviation was not reported in the published article on RCT, it was estimated either from the range or p-value or 0.5 was added in the cell frequency assuming the same variance in both the groups which might not be true in all the cases. The estimate of the difference between both techniques was pooled, depending upon the effect weights in results determined by each trial estimate variance.

### Quality of Analysis

**FIGURE 1:**
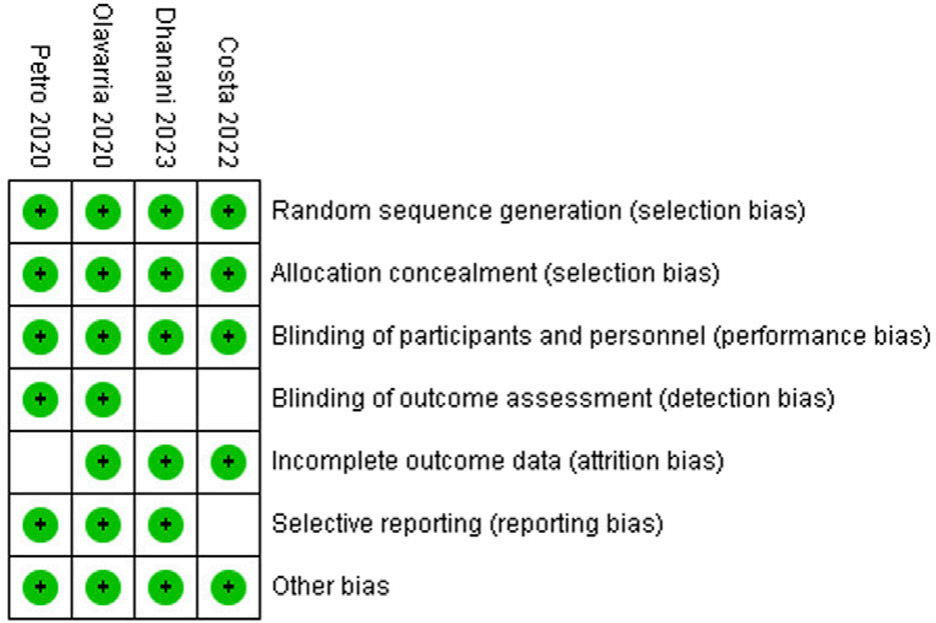
Risk of bias summary: review authors’ judgements about each risk of bias item in included trials.

**FIGURE 2:**
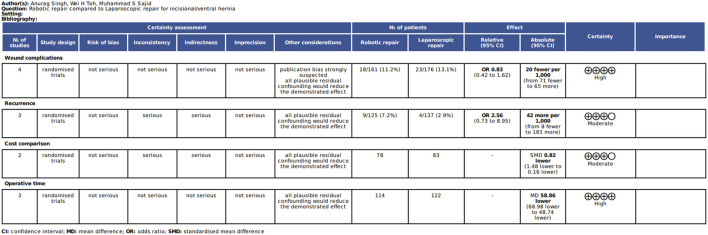
Evidence and summary of findings in accordance with the GRADEpro.

The quality of the included RCTs was assessed by using various reported tools including the tool provided by the Cochrane Collaboration [16–18]. The quality of included studies is given in Table 1 and depicted in Figures 1, 2.

**TABLE 1:** Quality of the included randomised control trials.

Study	Randomization technique	Concealment	Blinding	Intention to treat analysis	Ethical approval	Registration number	Power calculation
Costa 2022 [19]	Computer generated	Sealed envelopes	Single blinded	NR	Approved	NCT03283982	NR
Dhanani 2023 [20]	Computer generated	Sealed envelopes	Single blinded	NR	Approved	NCT03490266	Reported
Olavarria 2020 [21]	Computer generated	Sealed envelopes	Multi-blinded	Reported	Approved	NCT03490266	Reported
Petro 2020 [22]	Block randomisation	Concealed	Single blinded	Reported	Approved	NCT03283982	Reported

NR, Not reported.

### Selected Endpoints for Analysis

The recurrence of VH at the end of the follow-up period was considered the primary endpoint for this systematic review. The criteria to diagnose the VH recurrence included the symptomatic presentation of the patient with a recurrent lump at the site of previous surgery, clinical assessment by a senior surgeon and the reporting of the radiological diagnostic investigation to confirm it. The secondary endpoints were the surgical site complications, duration of operation, length of hospital stay and the cost of LVHR as well as the cost of RVHR.

## Results

**FIGURE 3:**
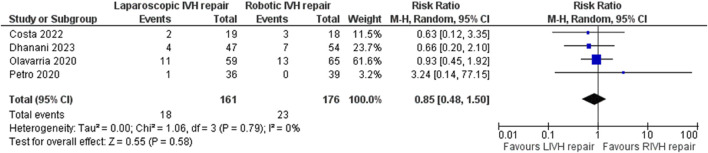
PRISMA flowchart showing literature search outcomes.

The primary search of the standard medical databases yielded 22 potential includable studies in this systematic review. After going through the various stages of screening, 18 trials were excluded due to the reasons given in the PRISMA flowchart (Figure 3).

### Methodological Quality of Included Studies

The reported quality variables in the included RCTs used to assess their strength of published evidence is summarized in Table 1. The randomization technique used in the included RCTs was computer generated in all RCTs [19–22]; the concealment was done using sealed envelopes in three included RCTs [19–21]; single blinding was reported in three RCTs [19, 20, 22] and multi-blinding [21] was reported in one included RCT. All included studies reported ethical approval and were registered before the conduction of the trial. Power calculation was done and reported in three studies [20–22].

### Characteristics and Demographics of Included Studies

Four RCTs [19–22] on 337 patients were included to study for perioperative outcomes and cost comparison. A one year-follow up of Petro, et, al; was used to study the recurrence rate as well [23]. One included RCT was reported from Brazil [19] and three reported RCTs were from the USA [20–22]. The characteristics of the included RCTs are presented in Table 2 and the treatment protocols used in the included studies are presented in Table 3.

**TABLE 2:** Demographics of the included studies.

Study	Country	Type	Age (mean ± SD) (Years)		Gender (female %)		Follow up duration
			Laparoscopic	Robotic	Laparoscopic	Robotic	
Costa 2022 [19]	Brazil	RCT	59.7 ± 12.7	65.2 ± 10.8	61.2	68.4	2 years
Dhanani 2023 [20]	United States	RCT	48 ± 13	50 ± 13	63	74	2 years
Olavarria 2020 [21]	United States	RCT	48 ± 12.9	50.1 ± 13.3	63	74	5 years
Petro 2020 [22]	United States	RCT	55 ± 8.18	56 ± 14.84	58	41	30 days

RCT, Randomised control trial; SD, Standard deviation.

**TABLE 3:** Treatment protocol among the included studies.

Study	Laparoscopic ventral hernia repair	Robotic ventral hernia repair
Costa 2022 [19]	• Hernia type - incisional hernia following laparotomy for abdominal malignancy • Mesh securing technique - intraperitoneally with 5 cm overlap • Type of mesh - Macroporous mesh • Hernia defect width - (mean ± SD) 8.9 ± 5.6 cm	• Hernia type - incisional hernia following laparotomy for abdominal malignancy • Mesh securing technique - intraperitoneally with a 5 cm overlap • Type of mesh - Macroporous mesh • Hernia defect width - (mean ± SD) 12.1 ± 5.3 cm
Dhanani 2023 [20]	• Hernia type – Primary, recurrent and incisional • Mesh securing technique - intraperitoneally with trans-fascial sutures and circumferential single/double permanent tacks • Types of mesh - mid-density coated polypropylene • Hernia defect width - median (IQR): 3 (1–4.5) (cm)	• Hernia type - Primary, recurrent and incisional • Mesh securing technique - intraperitoneally with running 2-0 barbed PDS circumferentially • Types of mesh - mid-density coated polypropylene • Hernia defect width - median (IQR): 3 (2,5) (cm)
Olavarria 2020 [21]	• Hernia type - Primary, recurrent and incisional • Mesh securing technique - intraperitoneally with trans-fascial sutures and a circumferential double crown of permanent tacks, defect closed with 0 polydioxanone sutures • Types of mesh - mid-density hydrogel adhesion barrier-coated polypropylene • Hernia defect width - Median (IQR): 3 (1–4.5) (cm)	• Hernia type - Primary, recurrent and incisional • Types of mesh - mid-density hydrogel adhesion barrier-coated polypropylene • Mesh securing technique - intraperitoneally with 2-0 PDS, defect closed with locking barbed 0 polydioxanone sutures • Hernia defect width - Median (IQR): 3 (2–5) (cm)
Petro 2020 [22]	• Hernia type - Primary, recurrent and incisional • Mesh securing technique - circumferentially with 4 permanent trans-fascial sutures followed by a double crown of permanent tacks, the defect was closed with the figure of 8 stitches using 0 monofilament permanent suture • Types of mesh - Barrier-coated monofilament polypropylene Hernia defect width - Median (IQR): 4 (2–5) (cm)	• Hernia type - Primary, recurrent and incisional • Mesh securing technique - circumferentially with 3/0 monofilament absorbable self-locking sutures, the defect was closed using 0 monofilament permanent suture • Mesh was secured Types of mesh - Barrier-coated monofilament polypropylene • Hernia defect width - Median (IQR): 3 (2.5–5) (cm)

SD, Standard deviation; PDS, Polydioxanone suture; IQR, Inter-quartile range.

### Primary Outcome Analysis

**FIGURE 4:**
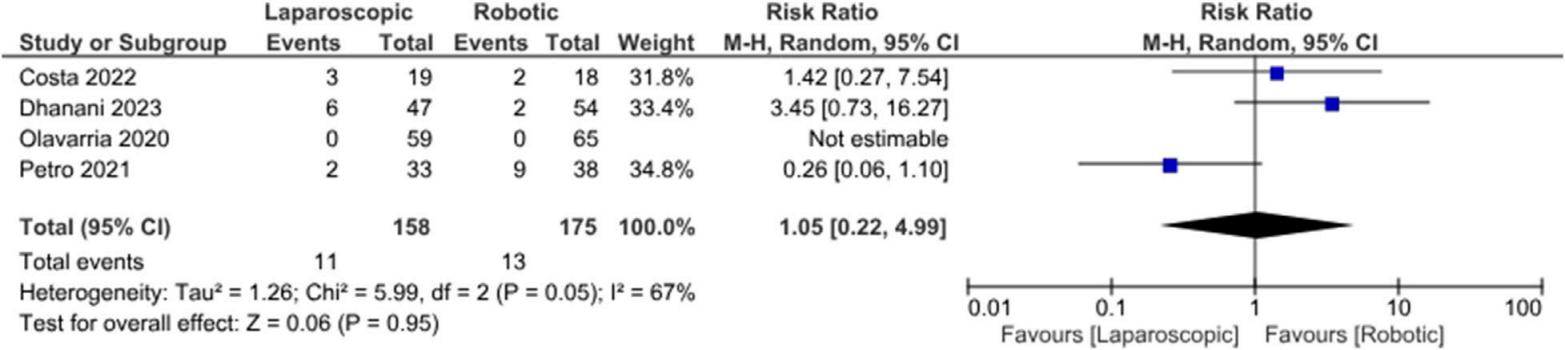
Forest plot showing the risk of recurrence in LVHR versus RVHR group. The outcome is presented as a risk ratio with a 95% confidence interval.

In the random effects model analysis, the incidence of VH recurrence was statistically similar between groups of laparoscopic versus robotic groups [RR 1.05, 95%, CI (0.22, 4.99), Z = 0.06, P = 0.95; Figure 4]. There was moderate heterogeneity among included RCTs (Tau^2^ = 1.26; Chi^2^ = 5.99, df = 2; (p = 0.05; I^2^ = 67%).

### Secondary Outcomes Analysis

**FIGURE 5:**
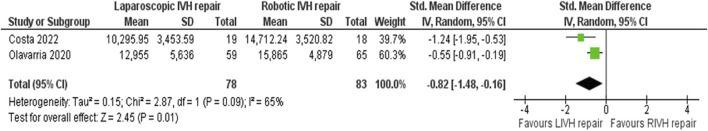
Forest plot showing the risk of surgical site complications in LVHR versus RVHR group. The outcome is presented as a risk ratio with a 95% confidence interval.

**FIGURE 6:**
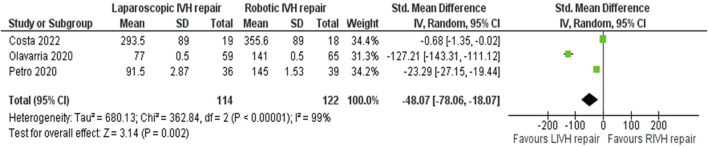
Forest plot showing the duration of operation in LVHR and RVHR cohorts. The outcome is presented as a standardised mean difference with a 95% confidence interval.

**FIGURE 7:**
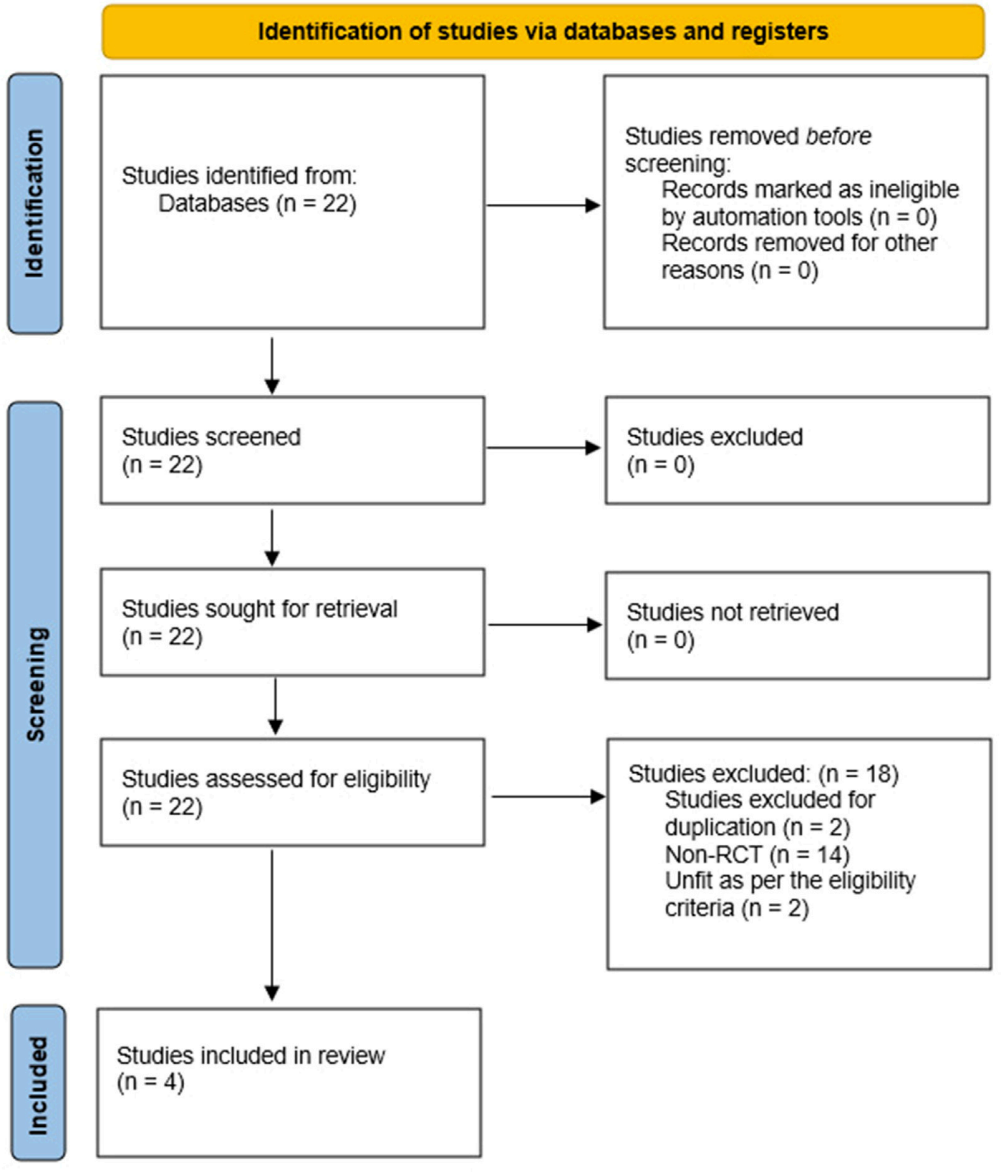
Forest plot showing the operative cost in LVHR and RVHR cohorts. The outcome is presented as a standardised mean difference with a 95% confidence interval.

In the random effects model analysis, the risk of surgical site complications (surgical site infection, seroma formation, wound break down, slow wound healing, failed wound healing) was statistically similar between LVHR group and RVHR group and there was no heterogeneity [RR, 0.85, 95%, CI (0.48, 1.50), Z = 0.55, p = 0.58; Figure 5], (Tau^2^ = 0.00; Chi^2^ = 1.06, df = 3; (p = 0.79; I^2^ = 0%). The duration of operation was shorter in patient undergoing LVHR compared to RVHR indicating superiority of laparoscopic approach over robotic approach [SMD -48.07, 95%, CI (−78.06, −18.07), z = 3.14, p = 0.002; Figure 6]. However, there was statistically significant heterogeneity among included studies on the calculation of this variable (Tau^2^ = 680.13; Chi^2^ = 362.84, df = 2; (p = 0.00001; I^2^ = 99%). In addition, LVHR was associated with reduced cost compared to RIVR in the random effects model analysis [SMD 0.82, 95%, CI (−1.48, −0.16), z = 2.45, p = 0.01; Figure 7]. However, there was statistically significant heterogeneity among included RCTs (Tau^2^ = 0.15; Chi^2^ = 2.87, df = 1; (p = 0.09; I^2^ = 65%).

## Discussion

Based upon the findings of this systematic review, LVHR seems to have the advantage over RVHR in terms of shorter duration of operation, lower cost of the procedure and equivalent efficacy for surgical site complications and hernia recurrence. RVHR failed to prove any clinical advantage over LVHR. Although these are very conclusive findings, however, this conclusion should be taken cautiously because of the paucity of RCTs with fewer patients undergoing VH repair.

### Comparison With Existing Literature

A previously published systematic review in 2023 [24] compared LVHR against RVHR and reported patient-related outcome measures. It was concluded that the available data on laparoscopic and robotic primary ventral hernia repair was scarce, and it was highly heterogeneous, thus making it difficult to assess the superiority of either approach in the management of VH. The current study provided evidence generated from the summated outcome of four RCTs and concludes that RVHR does not seem to have any proven clinical advantage over LVHR. Another published systematic review in 2020 [25] reported perioperative outcomes in a group of patients undergoing LVHR versus RVHR. The results of this review suggested that RVHR maintained some of the advantages of laparoscopic surgery and might provide an additional advantage of reduced hernia recurrence risk. This may well be explained by the ability to perform a more complex hernia repair with robotic assistance secondary to the ease of closure of the fascial defect. Whereas the current study analysed RCTs only and failed to demonstrate the previously reported advantage of the robotic approach. Several comparative trials have been reported with diverse outcomes [26, 27] and without any conclusive recommendations.

### Limitations

There was significant methodological and clinical diversity among included trials indicating heterogeneity. This study is based upon the findings of four RCTs on 337 patients and due to this reason, these findings cannot be generalised. The inclusion criteria were also different and patients with incisional hernia and primary ventral were jointly recruited in both limbs of trials which can potentially contribute to the biased outcome. The size of the hernia defect was reported in all studies, but it was of different size. The duration of follow-up in included RCTs varied from 1 year to 5 years which seems to be insufficient for the accurate estimation of the recurrence rate of hernia. Power calculations and intention to treat analysis were also not reported in the two included RCTs.

### Future Implications

A major multicentre RCT is mandatory to validate the findings of the current study before drawing a stronger conclusion about the advantages of the robotic approach in the management of VH. Trials on primacy ventral hernia and incisional hernia should be conducted separately to assess which group of hernia can benefit more from either approach. The patient recruitment criteria should be strict in terms of hernia defect size, type of mesh used, and technique of mesh fixation used to reduce methodological diversity in the RCTs. Trials should be conducted using a gold standard radiological diagnostic tool to detect clinical and subclinical recurrence for accurate measurement of primary outcome.
